# Using Non-Invasive Monitoring Technologies to Capture Behavioural, Physiological and Health Responses of Dairy Calves to Different Nutritional Regimes during the First Ten Weeks of Life

**DOI:** 10.3390/ani9100760

**Published:** 2019-10-02

**Authors:** Gillian Scoley, Alan Gordon, Steven Morrison

**Affiliations:** 1 Agri-Food and Biosciences Institute, Hillsborough BT26 6DR, Northern Ireland, UK; steven.morrison@afbini.gov.uk; 2 Institute for Global Food Security, School of Biological Sciences, Queen’s University Belfast, Belfast BT7 1NN, Northern Ireland, UK; 3 Agri-Food and Biosciences Institute, Newforge Lane, Belfast BT9 5PX, Northern Ireland, UK; alan.gordon@afbini.gov.uk

**Keywords:** dairy calves, welfare, behaviour, health, physiology, monitoring technologies

## Abstract

**Simple Summary:**

Recent research has debated the effects of milk and forage feeding regimes in the first weeks of life on the future performance of dairy calves. However, little is known about how feeding regime can affect behavioural and physiological responses, which have the potential to impact on calf health and well-being. Traditional methods of assessing calf health and welfare such as behavioural observations and blood sampling can be time consuming and impractical for producers and invasive for animals involved. Developments in technology have increased the availability of on-farm non-invasive devices which allow automatic and remote collection of behavioural and physiological data linked to animal health and welfare. This study aimed to use devices to measure lying behaviour, heart rate, heart rate variability and infrared temperature of calves offered high or low levels of milk replacer and different types of forage throughout the first ten weeks of life. Calves displayed changes in lying behaviour and heart rate variability as a result of changes in milk replacer feeding frequency. Additionally, infrared temperature changes were detected during periods of vaccination which corresponded with a rise in core body temperature. Results have highlighted that these sensors can provide important and useable data regarding overall calf well-being on commercial farms.

**Abstract:**

This study aimed to examine the use of non-invasive monitoring technologies as a means of capturing behavioural, physiological and health responses of calves allocated to different nutritional regimes. Seventy-four Holstein Friesian calves were individually penned and allocated to receive either high (HML) or conventional (CML) milk replacer (MR) levels between 5–70 days of age. Additionally calves were allocated to one of four forage treatments: (i) chopped straw offered between 14–70 days of age (CS14), (ii) chopped straw offered between 56–70 days of age (CS56), (iii) grass silage offered between 56–70 days of age (GS56), and (iv) no forage in the pre-wean period (NF). A representative sample of calves from each treatment were fitted with activity sensors and heart rate monitors throughout the experimental period to examine lying behaviour and heart rate variability, respectively. Thermal images of the eye and rectal area of each calf were taken 5 days/week between 5–77 days of age. Faecal and respiratory scoring of each individual calf was carried out on a daily basis throughout the experimental period. Milk replacer feeding level had limited effects on measures of calf health, although HML calves tended to have an increased likelihood for receiving treatment for scour than CML calves. Daily lying time (min/d) was lower in HML calves following reduction in MR feeding frequency at 43 days of age and weaning at 71 days of age when compared with CML calves. Additionally, HML calves displayed a lower heart rate variability following weaning, this suggestive of increased stress load. There were limited effects of forage treatment, however, CS14 calves displayed a greater daily lying time following MR step-down at 68 days of age, this potentially indicating increased rumination. Results of the present study highlight the benefits of using remote monitoring technologies as a means of detecting behavioural and physiological changes as a result of nutritional management strategy in individually housed dairy calves.

## 1. Introduction

Traditional calf feeding programs have involved providing calves with restricted levels of milk or milk replacer (MR) as a means of encouraging starter intake and thus facilitating earlier weaning and rumen development [1]. However, restricted milk feeding programs have also been linked with low live weight gains [2] and increased abnormal non-nutritive feeding behaviours suggestive of a decrease in calf welfare [3]. In recent years there has been growing interest in providing calves with higher levels of MR, which has been positively associated with improved growth rates in early life [4], reduced age at first calving [5] and increased first lactation milk yield [1]. However, providing greater volumes of milk or MR can result in reduced starter feed intake prior to weaning, [6] which may impede rumen development [7]. Gradual weaning programs have previously been suggested as a means of encouraging starter intake in calves offered high levels of milk [8]. However, as reported by de Passille, et al. [9], calves fed higher milk levels may still display lower total DMI than those fed conventional levels of milk following complete milk withdrawal despite the adoption of a gradual weaning program, which could impact on overall calf performance and welfare in the post-weaning period. Recently, Imani, et al. [10] reviewed the varying effects of forage provision on calf intake and performance during the transition from a liquid to a solid feed diet. Although results of previous research have been inconclusive, both Castells, et al. [11] and Khan, et al. [12] reported that forage provision improved feed intake and was beneficial for rumen development in calves offered conventional and increased levels of milk, respectively. Provision of forage has been shown to affect lying and feeding behaviour in calves offered high levels of MR [13]. However, little is known about the interactive effects of milk replacer level and forage inclusion on calf activity, which could have a potential effect on calf welfare.

Insufficient nutrition in the pre-wean period can impact negatively on immune function and increase the potential for susceptibility to infectious disease [14], it could be expected that providing increased levels of MR would result in fewer incidences of ill-health. However, results of previous research have been varied. Several authors have reported either improvements or no differences in health scores of calves offered increased levels of MR [4,15,16], whereas Quigley, et al. [17] reported increased incidence of diarrhoea and number of veterinary treatments in calves fed greater volumes of MR. Previous research has suggested that ill-health in early calf-hood can impact negatively on future performance [18] and that morbidity and mortality from disease is increased in the presence of physical and psychological stressors [19]. As indicated by Khan, et al. [20], neonatal calf feeding regime can have an extensive impact on performance, behaviour, health and welfare. Previous research has also provided evidence of long-term consistencies in individual differences of behavioural and physiological response to acute stressors [21], this suggesting that experiences in early calfhood could impact development of future coping strategies. As such, there is a need to examine if nutritional regime can impact on behavioural and physiological responses to management strategies commonly encountered in the first months of life.

Recently, Peli et al [22] reported on the neurochemical effects of stress related to intake of different diets in veal calves. The study described an increase in oxytocin production in veal calves offered either a MR only or MR plus concentrate diet (Control diet) when compared to calves offered a diet of MR, concentrate and hay. These results suggest that stress related to different diets can impact on measureable immunoreactive parameters and highlights the need for further examination of how nutritional regimes can impact on calf health and welfare. However, traditional methods of monitoring calf behaviour and reaction to stressors can be labour intensive (e.g., visual observations) and invasive (e.g., blood sampling, immunohistochemical methods). Recently there has been increasing interest in employing the use of remote-sensing technologies such as activity and heart rate monitors which could allow the non-invasive measurement of behavioural and physiological responses to stressors on an individual basis and provide a more comprehensive indicator of calf wellbeing [23,24]. Within this study the primary objective was to examine behavioural and physiological responses of calves to milk feeding level using remote monitoring technologies applicable to on-farm use. It was hypothesized that reduction in MR feeding frequency and weaning would likely be considered a stressor, particularly in those calves offered higher levels of milk replacer, and would provoke a measureable behavioural and physiological response. A further objective was to examine the impact of forage inclusion at different ages on calf activity and if there was an interactive effect with MR feeding level. 

## 2. Materials and Methods 

This study was conducted at the Agri-Food and Bioscience Institute (AFBI) research farm in Hillsborough (54°27′ N; 06°04′ W). The study commenced on 16th February 2016 and ended on 10th July 2016. The study described in this manuscript was approved by the Agri-Food and Biosciences Institute Animal Welfare and Ethical Review Body and all procedures and treatments were conducted under a license from the Department of Health, Social Services and Public Safety for Northern Ireland in accordance with the Animals (Scientific Procedures) Act 1986.

### 2.1. Animals

Seventy-four Holstein Friesian calves (39 females and 35 males) born between 16th February and 11th April 2016 were allocated to the study following weighing at ≤12 h of age which was used as the birth weight (40.8 ± 5.9 kg). All calves received 3 L colostrum via oesophageal feeding tube or teated bottle before transfer to the calf-rearing unit. Single point blood samples were taken via jugular venipuncture using 10 mL clot activated serum separation vacutainer tubes (BD, Plymouth, UK) at 24–36 h old to allow determination of calf immune status using the zinc sulphate turbidity (ZST) technique, as described by McEwan et al. [25]. All calves allocated to the study were determined to have a ZST value of >20 units, which is indicative of successful passive transfer of immunity [25]. 

### 2.2. Treatments and Experimental Design

Calves were individually penned in order of birth and allocated to treatment in a 2 (milk replacer feeding level) × 4 (forage provision) factorial design (Table 1). Milk replacer levels provided were either high (HML) or conventional (CML) (Table 2). Forage sources were provided at four different stages over the experimental period, with treatments designated as: (i) chopped straw (~4 cm in length) offered between 14 and 70 days of age (CS14), (ii) chopped straw offered between 56 and 70 days of age (CS56), (iii) grass silage offered between from 56 days of age (GS56), and (iv) no forage provided in the pre-wean period (NF). No forage source was provided prior to the commencement of treatments. Calves from all treatments were provided with grass silage as the sole forage source from 71 days of age until the end of experiment at day 84. 

### 2.3. Housing, Feeding and Management 

Individual pens (1m × 1.8m) were constructed using hurdles within two open ended concrete walled and floored housing blocks. A base layer of woodchip was added to the floor of each pen with sawdust bedding material added on top and replenished on a daily basis. Within these pens, calves had visual contact with all other calves within the housing block and nose-to-nose contact with calves from neighboring individual pens. Upon arrival to the calf rearing accommodation at ≤12 h of age all calves were fitted with jackets which remained on until calves reached 29 days of age. Calves remained in individual pens until the end of study at day 84.

Liquid diets were offered via a teated bucket twice a day on days 1 to 4. On days 1 to 3 each feed consisted of 2 L colostrum/transition milk, and on day 4 each 2L feed consisted of a mixture of half transition milk/half whey-based milk replacer. From 5 to 70 days of age, calves were offered milk replacer at a rate of 150g/L. Further details of milk replacer feeding regimes are provided in Table 2. Where calves were offered milk replacer 3 times daily, feedings occurred at 7:00–9:00 h a.m., 12:00–14:00 h p.m. and 16:00–18:00 h p.m. Where offered milk replacer twice or once daily the second or second and third feeds were removed respectively. Concentrate solid feed and fresh water were provided on an ad libitum basis from 5 days of age. Details of the chemical composition of feedstuffs offered throughout the study are reported in Table 3. Calves were vaccinated with Bovilis Bovipast RSP (MSD Animal Health, Milton Keynes, UK) at 2 weeks of age with a secondary dose given 4 weeks later at which point calves were also vaccinated with Bovilis IBR Marker Live (MSD Animal Health, Milton Keynes, UK). 

### 2.4. Data Collection

#### 2.4.1. Intakes and live weight

Individual daily milk replacer and concentrate intakes were recorded on a daily basis from 5 days of age. Individual water intake was recorded 5 days/week from 5 days of age. Forage intake was recorded on a daily basis where applicable. Live weight was recorded at birth as well as weekly according to individual calf age between days 7 and 84 (Tru-Test Eziweigh 5, Auckland, New Zealand). These results will be reported elsewhere.

#### 2.4.2. Calf Health

Faecal consistency was qualitatively scored on a daily basis following morning feeding time using the scale of 1 = normal 

Consistency, 2 = slightly liquid consistency, 3 = moderately liquid and 4 = primarily liquid consistency [17]. A calf was recorded as having scour when the score was greater than two. Respiratory disease scoring was carried out on a daily basis using the University of Wisconsin-Madison method [26]. This involved scoring calves on three visual aspects including; eyes, ears, and nasal discharge, the presence or absence of a cough and rectal temperature. Core body temperature was taken using a digital rectal thermometer (Model FT09, Beurer UK Ltd., Golborne, UK) on a daily basis between 5 and 77 days of age. Each aspect received a score from 0 to 3, with 0 representing normal and 3 the most severely affected with the overall respiratory score derived from the cumulative score of each aspect. A cumulative respiratory score of ≥5 was considered to indicate a bovine respiratory disease complex event. Faecal and respiratory scoring was carried out by a trained technician on an individual basis. Cases of calf ill health were recorded and treated according to predefined protocols produced in consultation with a veterinarian. 

#### 2.4.3. Thermal Imaging

Thermal images of each calf were taken 2 h post AM feed 5 days/week between days 7 and 77. Images were taken by the same trained operator using a calibrated FLIR E8 camera (FLIR Systems UK, Kent, U.K.) and were of the right eye (plus a 1 cm area surrounding the eye) and the anus (plus a 1cm area surrounding the anus). Images were taken at a consistent distance (~0.5 m) and angle (~90°) whilst the calf was standing. For purposes of standardisation, images were taken within the individual pens prior to introduction of any potential stressors such as weighing or blood sampling. As calves quickly became habituated to the process, very limited handling from the camera operator was required. Images were processed using FLIR^®^ software (FLIR Systems UK, Kent, U.K) with maximum, average and minimum temperature of images recorded. Ambient temperature and relative humidity (RH) within the rearing accommodation were recorded using a calibrated EBI 20-TH data logger (ebro Electronic, Ingolstadt) throughout the experimental period. Average temperature and RH values obtained during the time period when images were taken were entered into the software program during image processing to allow for atmospheric changes during the sampling period. Analysis of eye images focused on the medial, posterior, palpebral border of the lower eyelid and the lacrimal caruncle as these have been found to be the area of most consistent temperature [27].

#### 2.4.4. Lying Behaviour 

A total of 28 calves, balanced across milk replacer and forage treatments were fitted with IceRobotics^®^ IceQube^®^ automatic activity sensors (IceRobotics Ltd., Edinburgh, Scotland, UK) at 5 days of age and remained on calves throughout the experimental period. Sensors were affixed to the lateral side of the right rear leg in accordance with current best practice [28,29,30] with sampling rate kept at the default (1 s) interval. Once removed, sensors were downloaded and an optimal correction filter [30] which removed sensor recordings lasting ≤ 8 s was applied to data prior to analysis. 

#### 2.4.5. Heart Rate Monitors 

A total of 36 calves, balanced across milk replacer and forage treatments, were fitted with a Polar Equine RS800CX Science (Polar Electro UK Ltd, Heathcote Way, Warwick, UK) heart monitor on days −2, −1, 0, +1 and +2 where day 0 = day of 1st vaccination, day of 2nd vaccination and days 29, 43, 57, 68 and 71 of age. These ages represented calf jacket removal (d29), changes in milk feeding frequency and quantity (d43, 57 and 68) and weaning (d71). Monitors were fitted 1–2 h post AM feed and remained on calves for 1–2 h to allow data capture during the period when calves were undisturbed and most likely to be resting. Sampling was conducted at the same time each day to help decrease any effects of circadian rhythms. Electrode gel (Spectra 360 Electrode Gel, Parker Laboratories Inc., New Jersey, USA) was applied to ensure contact between the calf and monitor [31]. The time period from which data for analysis was selected was between 5–10 min [32]. Following data selection, recordings were processed using both the Artiifact and Polar software [33]. Preliminary error correction of the data set was conducted using the Polar software settings as described by Clapp, et al. [31], with any data set requiring over 5% error correction rejected [34]. Following error correction, the data set was further processed using Artiifact software with resultant root mean squares of successive differences (RMSSD) in inter heart beat intervals (IBI) and heart rate (HR) used in the analysis [35].

### 2.5. Statistical Analysis

All data were analysed using GenStat^®^ (version 16.2, VSN International Ltd.). All statistical models included sex as a fixed effect and birth weight as a covariate unless otherwise stated. A probability of *p* < 0.05 was selected as the level of significance, and where data was significant it was subjected to Fisher’s protected least significant difference (PLSD) test. 

As variation in daily faecal and total respiratory scores was low, they were averaged on a weekly basis for the first four and complete ten weeks of the experimental period, respectively. Mean weekly scores were then fitted to a repeated measures residual maximum likelihood estimation (REML) mixed effects model with fixed effects and associated interactions of milk replacer level and forage treatment. Likelihood of an animal receiving treatment for scour, pneumonia or a high temperature with signs of respiratory illness was analyzed by modelling of binomial proportions using logistic regression. Fixed effects of sex, milk level, forage treatment and interactions of milk level and forage treatment were fitted individually to the model and back transformed means are reported.

Thermal imaging and rectal temperature data were assigned to four age bands; d7–21 to represent the period over which first vaccination occurred, d22–42 to represent the period following first vaccination and prior to first milk step-down of accelerated calves, d43–56 to represent the period following first milk step-down and the time during which second vaccination occurred and d57–77 to represent the period over which milk step downs and weaning occurred. Data were fitted to a repeated measures REML model with fixed effects and associated interactions of milk replacer level, forage treatment and time. Ambient temperature was included as a covariate. These data were also fitted to a repeated measures REML model with fixed effects and associated interactions of milk replacer level, forage treatment, time and method of temperature assessment. Correlation coefficients between each of the three temperature methods were determined by linear regression using the predicted means obtained through the repeated measures analysis.

Daily and hourly lying time along with number and duration of lying bouts and heart rate and heart rate variability data were fitted to the same repeated measures REML model as described above. Results pertaining to hourly lying behaviour are presented in Supplementary data Figure S1. Within the lying behaviour and heart rate analysis, where results refer to the period of time following a management event (e.g., MR step-down), a baseline covariate derived from averaging data from the 5 (activity sensors) or 2 (heart rate monitors) days prior to the event was included within the model.

## 3. Results

### 3.1. Calf Health

Calves offered high (HML) levels of milk replacer had an increased average faecal score between days 14–20 when compared to calves offered conventional (CML) levels of milk replacer (Table 4; *p* = 0.010). There was no effect of milk replacer level or forage treatment on average faecal score between days 5–13, 21–27 or 28–34, or of forage treatment between d14–20 (Table 4; *p* > 0.1). No faecal scores above 1 were recorded after 34 days of age. CML calves had an increased average respiratory score between days 49–55 when compared to HML calves (Table 4; *p* = 0.022), however, despite the observed increase, average respiratory scores across both treatments remained below the threshold deemed indicative of bovine respiratory disease. There was no effect of forage treatment on respiratory score (Table 4; *p* > 0.1). There was no effect of milk level offered on the probability of receiving treatment for pneumonia (*p* = 0.189) or pyrexia plus respiratory type symptoms (*p* = 0.599). Calves offered chopped straw at 56 days of age (CS56) had an increased likelihood of receiving treatment for pneumonia (*p* = 0.008), however, no interactive effects of MR level and forage treatment were observed (*p* = 0.831). An increased likelihood of receiving treatment for scour was observed in HML calves (*p* = 0.059) with values of 0.47 and 0.26 for HML and CML calves respectively. There was no significant effect of forage treatment (*p* = 0.130) or MR level × forage treatment (*p* = 0.196) on likelihood of receiving treatment for scour.

### 3.2. Calf Temperature Assessment

**Figure 1:**
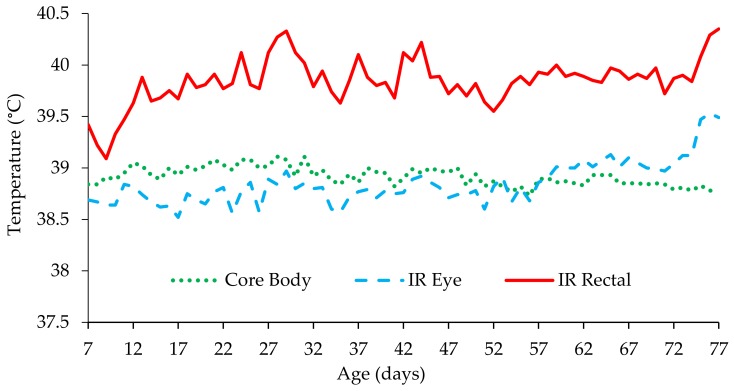
Core body temperature as measured via digital rectal thermometer, IR eye and IR rectal temperature of calves between 7 to 77 days of age.

**Figure 2:**
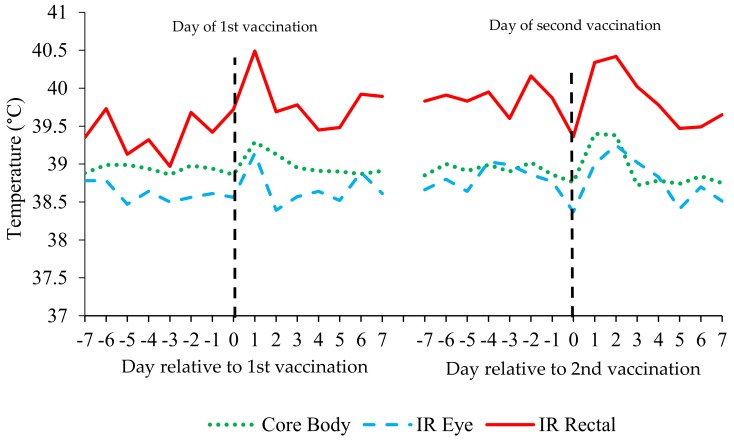
Core body temperature as measured via digital rectal thermometer, IR eye and IR rectal temperature of calves over the periods of 1st and 2nd vaccination.

There were no effects of MR feeding level or forage treatment on core body, infrared (IR) eye or IR rectal temperature between days 7–21, 22–42, or 43–56 (Table 5; *p* > 0.1). Average core body, IR eye and IR rectal temperatures recorded between d7–77 are presented in Figure 1. Core body temperature was 38.80 and 38.87 in HML and CML calves respectively between days 57–77 (Table 5; *p* = 0.057), however, there were no differences in IR eye or IR rectal temperature. There were no differences between MR or forage treatments in core body, IR eye or IR rectal temperature when data were analysed over the 7 days prior to and following calf jacket removal, step down in MR feeding frequency or quantity or weaning. Although unaffected by MR feeding level, there was an effect of day with an increase in all temperature parameters in the 2 days following 1st and 2nd vaccination (Figure 2; *p* < 0.001).

Calf temperature was affected by method of temperature assessment across all time periods. Positive correlations were found between core body and IR eye temperature when data were analysed between 7–21, 22–42, 43–56 and 57–77 days of age (Table 6). Positive correlations were also found between core body and IR rectal temperature between days 7–21, 22–42 and 57–77 and between IR eye and IR rectal temperature during days 22–42, 43–56 and 57–77 (Table 6). Correlations of 0.4, 0.6 and 0.39 were found between core body and IR eye, core body and IR rectal and IR eye and IR rectal temperatures respectively when analysed over the 7 days prior to and following removal of calf jackets (Table 6). Strong positive correlations were also found between methods of temperature assessment during the 7 days prior to and following 2nd vaccination (Table 6).

### 3.3. Activity Monitors

Daily lying time was increased in HML calves between days 36–42 (*p* = 0.017), and was characterized by an increased number (*p* < 0.001) of shorter (*p* = 0.002) lying bouts when compared with CML calves. During this period, calves offered chopped straw from 14 days of age (CS14) displayed an increased number (20.0 vs 15.4) of shorter (56.2 vs 71.6 min) lying bouts than those offered chopped straw at 56 days of age (CS56). However, despite this variation, there no was no significant impact of forage treatment on total daily lying time (Table 7; *p* = 0.306). Daily lying time was 40 min/d lower in HML calves in the period following their step down from three to two MR feeds at 43 days of age when compared with CML calves (Table 7; *p* = 0.021). There was no significant effect of MR feeding level on daily lying behaviour between d57–62 (Table 7; *p* > 0.05), however, there was an effect of forage treatment whereby CS56 calves had fewer lying bouts (Table 7; *p* = 0.028) than CS14 and GS56 calves (19.3 vs 22.0 vs 22.7). Lying behaviour was not significantly impacted by MR feeding level in the period following reduction of MR to 2L/day at 68 days of age (Table 7; *p* > 0.1), however, there was an effect of forage treatment whereby total daily lying time was higher (Table 7; *p* = 0.022) in CS14 calves when compared with GS56 and NF calves (1112 vs 976 vs 986 min/d). Daily lying time was higher in CML calves in the period following weaning at 71 days of age (Table 7; *p* = 0.026), lying bouts also tended to be in longer in these calves when compared to HML calves (Table 7; *p* = 0.098). There was no effect of forage treatment on lying behaviour during the post weaning period, however, there was an effect of MR level × forage treatment on lying bout duration (Table 7; *p* = 0.014), with bouts approximately 10.8 min shorter in HML calves receiving chopped straw at 56 days of age. Diurnal lying behaviour over periods of milk step-down and weaning is presented in Supplementary data Figure S1.

### 3.4. Heart Rate Monitors

There was no effect of MR feeding level on heart rate (HR) or heart rate variability (HRV) as measured by root mean square of successive differences (RMSSD) during the period following calf jacket removal at 29 days of age. This was also true in the periods following step down of HML calves from three to two and two to one MR meals/d at 43 and 57 days of age, respectively, and following reduction of MR level to 2L/day across treatments at 68 days of age. Both heart rate and RMSSD values were unaffected by MR treatment in the period following weaning at 71 days of age, however, there was an interactive effect of MR treatment and day, with HML calves displaying increased heart rate (Table 8; *p* < 0.001) and lower RMSSD (Table 8; *p* = 0.058) at 72 days of age. There was no significant effect of forage treatment on HR or RMSSD throughout the experimental period (Table 8).

## 4. Discussion

Responses to common management practices such as restricted feeding and weaning have previously been assessed using measures of calf activity, with technological developments meaning that standing and lying behaviour can now be recorded automatically [36]. De Paula Vieira, et al. [37] reported a decrease in lying time observed in limit-fed calves, and suggested that this was due to hunger. In the present study, daily lying time was reduced by 31 min/d in CML calves, and was characterized by a reduced number of shorter lying bouts between d36–42, the period prior to reduction in MR feeding frequency and volume for HML calves. As reported by Terré, et al. [7], calves offered lower levels of MR consume more concentrate. In the present study, and as will be reported elsewhere, average daily concentrate consumption between d5–42 was 190 g DM and 330 g DM for HML and CML calves, respectively (*p* < 0.001). As such it is likely that CML calves in the present study were spending more time standing to consume concentrate that HML calves. Calves offered chopped straw at 14 days of age (CS14) also displayed an increased number of shorter lying bouts during this period. Castells, et al. [11] reported differences in calf activity between calves offered alfalfa hay and those not provided with forage, it could be therefore that changes in behaviour observed in the present study were due to CS14 calves consuming the chopped straw. At 43 days of age, MR volume and feeding frequency was reduced in HML calves. During this period, lying time was reduced in these calves both in comparison to the pre-step down period and to that recorded in CML calves. A reduction in lying time was also observed in HML calves in the period following complete milk withdrawal. The decrease in lying time in HML calves is similar to that reported by Budzynska and Weary [28] and likely a sign of increased hunger following reduction in or withdrawal of MR. As indicated by de Passille, et al. [9], a decrease in overall DMI due to reduced solid feed intake has been observed in calves offered high levels of MR in the pre-wean period even when managed in a gradual weaning program. The decrease in lying time in HML calves during this period of milk step down and complete weaning could therefore be due to HML calves spending longer standing to consume solid feed as a means of addressing the reduction in available feed energy. During d68–70, CS14 calves displayed increased lying time when compared to GS56 and NF calves. Calves spend more time ruminating whilst lying down [11], as such it could be considered that rumen development was potentially more advanced at in calves offered forage at an earlier age and the increase in lying time was due to increased rumination. The lack of direct observations to distinguish specific behaviours such as feeding and rumination could be considered a limitation to the present study, future research could implement the use of calf appropriate rumination halters to allow feeding behaviours to be distinguished. However, the results do highlight how activity monitors could be used on-farm by producers to distinguish changes in behaviour as a result of changes in nutritional regime. Application of activity meters in group housed situations could also provide further information regarding the impact of feeding regime on diurnal behaviour of dairy calves.

Heart rate monitors have previously been shown to provide increased opportunities for non-invasive monitoring of physiological responses to stress [38]. An increase in heart rate (HR) and decrease the root mean square of successive differences (RMSSD), a time domain measure of heart rate variability (HRV), have been linked to increased stress load [39]. In the present study measures of HR and HRV were comparable across the pre-wean period, however there was a reduction in RMSSD in HML calves following weaning. As HR and HRV measurements were collected when the calves were at rest [39], this could indicate a potential increase in stress response and corresponds with the changes in lying behaviour observed in HML during this period. This again highlights that calves high levels of MR in the first weeks of life could display an increased stress response to withdrawal of MR when compared with calves previously fed a conventional level of MR. Adopting a more prolonged weaning approach whereby MR volume is reduced on a daily basis, such as that described by Sweeney, et al. [8], may improve concentrate intake in calves fed high levels of MR and thus reduce stress associated with MR withdrawal.

Ill-health in early calfhood can have a significant impact on animal behaviour, welfare and productivity, all of which can affect the economic efficiency of dairy operations [40,41]. Enteric and respiratory diseases are the most common causes of morbidity and mortality in the first weeks of life, prevalence and severity of which can be affected by housing conditions, colostrum intake and feeding management [42]. Providing calves with milk or milk replacer at a level equivalent to 10% of calf birth weight per day in two portions is an accepted industry standard and is designed to encourage earlier intake of solid feed [17]. However, it has been suggested that restricted feeding can impact negatively on growth rates and immune function in calves and thus may potentially increase susceptibility to disease [43].

In the present study, it could have been considered that calves offered high (HML) levels of MR would have been less likely to suffer from ill-health when compared to those offered conventional (CML) levels of MR. However, comparable to that reported by Quigley, et al. [17], HML calves in the present study had increased faecal scores between d14–20 and were more likely to receive treatment for diarrhoea than CML calves. In the study by Quigley, et al. [17], calves fed either conventional or increased levels of MR were subject to transport stress and then housed in hutches where the bedding material had been contaminated with coronavirus. As increased incidence of morbidity and mortality was observed in calves provided with increased levels of MR in the study, this was directly apportioned to the feeding level, leading the authors to advise a cautionary approach if providing increased MR levels to calves under stress. Diaz, et al. [44] also reported increased faecal scores in calves offered increased levels of MR, however, this was considered to be as a result of the differences in total volume of feed and water consumed as opposed to disease. In the present study although faecal score was elevated in HML calves, it was below that considered indicative of neo-natal calf diarrhoea (NCD), suggesting that the increased scores were, as with Diaz, et al. [44], likely as a result of the fact that HML calves were receiving 5l/day more MR than the CML calves. Additionally, anecdotal evidence within the present study indicated that scour was milky in colour, reinforcing the consideration that the higher faecal scores in HML calves was due to the higher initial MR volume as opposed to infection. Further research could aim to examine if scour scoring systems could be modified or improved to take into account the presence of milky scour. The average respiratory score observed in CML calves between days 56–62, although increased in comparison to HML calves, was again below the threshold score considered to be indicative of BRD. These results indicate that under the management conditions described, calves can be fed either increased or conventional levels of milk replacer without severe impact on health throughout the pre-wean period. This may have been influenced by the fact that calves were individually housed which has been shown to reduce health problems [45]. However, the increased faecal scores in the first 3 weeks of life in HML calves could suggest that it may be advisable to delay increasing MR levels until after this period when calves are less susceptible to enteric disease [46]. 

One of the primary detectable physiological responses to infection and inflammation is a change in core body temperature, and as such it is often used as an indicator of ill-health [47]. In addition to illness, stress, a result of common management practices in early calfhood, can also result in changes in blood flow and thus heat production as a result of increased hypothalamic pituitary adrenal (HPA) axis activity [48]. Infrared thermography (IRT) has been indicated as a non-invasive and viable method of detecting these small changes in radiated temperature [49] and has been highlighted as a potential method for the early detection of ill-health [50]. In the present study, there was no difference in radiated temperature of the eye or rectal area, this suggesting that calves were able to maintain thermoregulation throughout the experimental period despite differences in MR feeding levels. This was particularly evident in the period following calf jacket removal, as it could have been anticipated that CML calves may have struggled to maintain thermoregulation when faced with the combined factors of reduced energy intake and lower ambient temperatures, however, no difference in temperature measurements were found. This could have been due to the fact that calves were 4 weeks of age at the time of calf jacket removal and, as such, were better able to withstand lower ambient temperatures [51]. At this age CML calves were also likely to be consuming greater amounts of solid feed [52] and thus nutritional energy was potentially sufficient to cope with increased heat production demands [53] as a result of exposure to lower ambient temperatures following calf jacket removal.

There is limited published research addressing the correlation between infrared (IR) temperature and core body temperature in very young calves. Although variable in the present study, correlation between IR eye temperature and core body temperature was within the range to that found in Jersey heifers by Salles, et al. [54] and comparable to the correlation between core body temperature and indwelling rumen bolus temperature reported by Knauer, et al. [55]. The reason for the variability in correlation is unknown and requires further investigation. However, it could be that IR temperature should be used as a standalone measure, wherein individual calves are used as their own control, particularly in situations when incidence of ill-health/pyrexia is low. The results of the present study suggest that IR temperatures were not affected by the MR feeding level. However, it may also be that the number of calves used in the experimental design was not sufficient to detect any differences as a result of feeding level. Additionally, there is limited information regarding the effects of feeding level on body temperature, an area requiring further investigation. Comparable to previous research [56], in the present study, IRT was able to detect the febrile response linked to vaccination. Again, this response did not differ between MR treatments, however, it does highlight the potential for IRT to be used in the detection of pyrexia associated with symptoms of disease and, if automated as per Schaefer, et al. [57], it could offer a less invasive option of continuous temperature assessment in young calves. 

## 5. Conclusions

Under the management conditions described in the present study, MR feeding level had limited effects on calf health and physiological responses to common management practices. However, changes in activity which could be linked with hunger as a result of conventional feeding levels and following reduction in MR volume in calves offered high levels of MR in early life were detectable using automatic activity sensors. Additionally, the higher lying time observed in CS14 calves between 68–70 days of age could point to increased rumination as a result of earlier forage introduction. This suggests that providing calves with a forage source in addition to concentrate feed could be beneficial to rumen development. In the present study, calves were individually housed which perhaps contributed to the low level of ill-health amongst calves. Calves were also monitored on a daily basis with a combination of traditional and novel indicators, this potentially resulting in earlier detection and treatment of ill-health, as such, future research should incorporate techniques described here into group housing systems. Results of the present study also provide evidence for the role of remote-sensing technologies within calf rearing systems. Using a range of traditional and novel methods increases the possibilities for collection of behavioural and physiological data. This will allow producers to obtain an overall indicator of calf well-being and responses to common management practices encountered in early life. Further research which aims to compare and correlate these technologies with hormonal, biochemical and immunological parameters could help in the development of early warning systems for calf health and welfare issues.

## Figures and Tables

**Table 1:** Number of calves allocated to each treatment.

Milk Replacer Level	Forage Provision			
	CS14 ^1^	CS56 ^2^	GS56 ^3^	NF ^4^
HML ^5^	11	9	10	9
CML ^6^	8	10	8	9

^1^ CS14 = Chopped straw provided from 14 days of age; ^2^ CS56 = Chopped straw provided from 56 days of age; ^3^ GS56 = Grass silage provided from 56 days of age; ^4^ NF = No forage provided during the pre-wean period; ^5^ HML = High milk level from 5 days of age; ^6^ CML = Conventional milk level from 5 days of age.

**Table 2:** Milk replacer feeding regime for calves in the first ten weeks of life.

Milk Replacer Feeding Level		Calf Age (days)			
		d5–42	d43–56	d57–67	d68–70
IML ^1^	Milk Replacer (g/day)	1350	900	450	300
	Milk Replacer (L/day)	9	6	3	2
	Daily feeding frequency	3	2	1	1
CML ^2^	Milk Replacer (g/day)	600	600	600	300
	Milk Replacer (L/day)	4	4	4	2
	Daily feeding frequency	2	2	2	1

^1^ IML = Increased milk level from 5 days of age; ^2^ CML = Conventional milk level from 5 days of age.

**Table 3:** Chemical composition of milk replacer, concentrate, chopped straw and grass silage offered to calves throughout the experimental period.

	Milk Replacer	Concentrate	Chopped Straw	Grass Silage
DM (%)	95.09	91.07	85.44	34.52
CP (% of DM)	23.31	19.17	3.03	16.50
NDF (% of DM)	-	24.90	84.99	45.84
ADF (% of DM)	-	11.76	50.38	27.67
Ash (% of DM)	7.87	6.42	5.39	10.57
GE (MJ/kg DM)	20.80	18.09	18.41	19.04

**Table 4:** Mean weekly faecal and respiratory scores of calves offered high or conventional levels of milk replacer.

	Milk Replacer (MR) Level		SED	*p*-Value		
	HML ^1^	CML ^2^		MR Level	Forage	MR × Forage
Faecal Score						
d5–13	1.21	1.14	0.055	0.257	0.105	0.409
d14–20	1.16	1.05	0.038	0.010	0.260	0.964
d21–27	1.03	1.01	0.015	0.266	0.235	0.678
d28–34	1.01	1.01	0.010	0.949	0.742	0.151
Respiratory Score						
d5–13	1.53	1.49	0.093	0.583	0.836	0.278
d14–20	1.63	1.73	0.093	0.283	0.780	0.480
d21–27	1.70	1.73	0.130	0.790	0.145	0.922
d28–34	1.62	1.54	0.128	0.540	0.686	0.902
d35–41	1.53	1.53	0.135	0.972	0.217	0.636
d42–48	1.48	1.65	0.127	0.180	0.576	0.467
d49–55	1.27	1.57	0.128	0.022	0.392	0.676
d56–62	1.29	1.44	0.098	0.125	0.288	0.050
d63–69	1.38	1.48	0.010	0.368	0.099	0.523
d70–77	1.35	1.50	0.092	0.123	0.452	0.270

^1^ HML = High milk level from 5 days of age; ^2^ CML = Conventional milk level from 5 days of age.

**Table 5:** Average core body, infrared (IR) eye and IR rectal temperature of calves managed under different nutritional regimes throughout the experimental period.

Time Period	Temperature Method	Milk Replacer (MR) Treatment		SED	*p*-Value			
		HML ^1^	CML ^2^		Forage	Day	MR	MR × Day
d7–21	Core body ^3^	38.97	38.93	0.042	0.656	0.028	0.386	0.302
	IR eye	38.76	38.63	0.089	0.959	0.797	0.204	0.834
	IR rectal	39.70	39.55	0.140	0.366	0.006	0.440	0.620
d22–42	Core body	38.99	38.95	0.051	0.275	0.166	0.430	0.711
	IR eye	38.81	38.72	0.085	0.776	0.300	0.332	0.847
	IR rectal	40.03	39.85	0.128	0.560	0.029	0.208	0.669
d43–56	Core body	38.86	38.93	0.051	0.234	0.496	0.157	0.124
	IR eye	38.78	38.81	0.093	0.769	0.389	0.890	0.967
	IR rectal	39.72	39.91	0.119	0.747	0.009	0.168	0.653
d57–77	Core body	38.80	38.87	0.037	0.105	0.837	0.057	0.844
	IR eye	39.11	39.09	0.071	0.820	<0.001	0.806	0.654
	IR rectal	39.89	39.97	0.095	0.840	0.014	0.253	0.729

^1^ HML = High milk level from 5 days of age; ^2^ CML = Conventional milk level from 5 days of age; ^3^ Core body temperature measured via digital rectal thermometer.

**Table 6:** Pearson correlation coefficients between methods of temperature assessment over specific age ranges and during the 7 days pre- and post-management events.

	Methods of Temperature Assessment					
	Core Body × IR Eye	*p*-Value	Core Body × IR Rectal	*p*-Value	IR Eye × IR Rectal	*p*-Value
Age Range						
d7–21	0.14	0.091	0.56	<0.001	NC1	0.473
d22–42	0.23	0.016	0.20	0.024	0.39	0.001
d43–56	0.17	0.077	0.11	0.137	0.19	0.070
d57–77	0.24	0.014	0.12	0.070	0.62	<0.001
Week pre-and post-event						
1st vaccination	0.20	0.007	0.10	0.140	0.27	0.002
2nd vaccination	0.39	0.007	0.62	<0.001	0.59	<0.001
Calf jacket removal	0.40	0.007	0.60	<0.001	0.39	0.008
1st step down	0.60	<0.001	0.11	0.122	0.37	0.010
2nd step down	NC^1^	0.516	NC^1^	0.342	NC^1^	0.748
3rd step down	NC^1^	0.627	NC^1^	0.900	0.46	0.003
Weaning	0.08	0.160	0.07	0.178	0.96	<0.001

^1^ NC = No correlation coefficient available as value outside lower bounds.

**Table 7:** Daily lying behaviour of calves managed under different nutritional regimes throughout the experimental period.

Management Event	Lying Behaviour Parameter	Milk Replacer (MR) Level		SED	*p*-Value					
		HML	CML		Day	Forage	MR Level	Day × Forage	Day × MR Level	Forage × MR Level
Pre-step-down d36–42	Lying bout frequency	19.4	16.1	1.32	0.879	0.003	<0.001	0.720	0.249	0.614
	Lying bout duration (min)	56.8	67.5	3.30	0.873	0.004	0.002	0.991	0.513	0.745
	Lying time/day (min)	1049	1018	13.1	0.549	0.306	0.017	0.960	0.974	0.403
1st MR step-down d43–48	Lying bout frequency	16.7	18.0	0.78	0.630	0.440	0.125	0.559	0.396	0.114
	Lying bout duration (min)	66.4	63.5	4.57	0.449	0.594	0.628	0.721	0.167	0.574
	Lying time/day (min)	1018	1058	16.5	0.325	0.889	0.021	0.535	0.930	0.093
2nd MR step-down d57–62	Lying bout frequency	21.8	20.3	1.09	0.069	0.028	0.113	0.298	0.528	0.472
	Lying bout duration (min)	47.6	53.0	3.61	0.039	0.147	0.097	0.354	0.698	0.309
	Lying time/day (min)	997	1022	30.5	0.983	0.361	0.430	0.878	0.190	0.192
3rd MR step-down d68–70	Lying bout frequency	22.0	22.8	1.61	0.877	0.666	0.671	0.788	0.537	0.882
	Lying bout duration (min)	49.8	46.9	3.41	0.393	0.983	0.434	0.921	0.577	0.165
	Lying time/day (min)	1041	1036	44.5	0.879	0.022	0.926	0.947	0.817	0.482
Weaning d71–76	Lying bout frequency	23.9	22.3	1.26	0.144	0.554	0.233	0.103	0.248	0.166
	Lying bout duration (min)	44.0	48.6	2.47	0.756	0.285	0.098	0.962	0.337	0.014
	Lying time/day (min)	996	1063	27.2	0.083	0.389	0.026	0.609	0.951	0.373

**Table 8:** Heart rate (bpm) and heart rate variability (RMSSD) of calves managed under different nutritional regimes in the first ten weeks of life.

Management Event		Milk Replacer (MR) Level		SED	*p*-Value			
		HML	CML		Day	Forage	MR	Day × MR
Calf jacket removal	RMSSD	25.0	31.4	8.51	0.339	0.410	0.383	0.716
	HR (bpm)	104.5	95.8	7.93	0.560	0.571	0.463	0.691
1st MR step down	RMSSD	31.0	23.9	5.29	0.995	0.065	0.151	0.215
	HR (bpm)	99.6	93.0	5.89	0.223	0.089	0.484	0.554
2nd MR step down	RMSSD	29.1	33.3	6.05	0.653	0.638	0.401	0.624
	HR (bpm)	92.0	93.4	3.44	0.269	0.341	0.640	0.860
3rd MR step down	RMSSD	19.0	29.2	4.81	0.639	0.657	0.128	0.106
	HR (bpm)	100.1	97.2	4.26	0.385	0.456	0.708	0.118
Weaning	RMSSD	22.6	23.2	5.73	0.711	0.717	0.688	0.058
	HR (bpm)	99.6	98.2	4.41	0.603	0.735	0.787	<0.001

## Supplementary Materials

The following are available online at https://www.mdpi.com/2076-2615/9/10/760/s1, Figure S1: Diurnal lying behaviour of calves offered increased (HML) or conventional (CML) levels of milk replacers over periods of milk step down and weaning. *p*-values represent the effect of milk replacer feeding level.
