# Supplementary material for: Using Non-Invasive Monitoring Technologies to Capture Behavioural, Physiological and Health Responses of Dairy Calves to Different Nutritional Regimes during the First Ten Weeks of Life
Source: Animals (Basel). 2019 Oct 2;9(10):760. doi: 10.3390/ani9100760 (PMC6827363; doi:10.3390/ani9100760)
Supplement: Supplementary file 1 [file animals-09-00760-s001.pdf]

Hourly lying behaviour

Calves allocated to the CML treatment tended to have reduced hourly lying times when compared with HML calves in the pre-step down period (Figure S1; P=0.067). HML calves tended to have reduced hourly lying times in the period following step-down from three to two MR feeds at 43 days of age (step-down 1) when compared with CML calves (P=0.066). There was no effect of MR level on hourly lying time in the period following reduction of MR to 2L/day at 68 days of age (step-down 3). Hourly (Figure S1; P=0.083) lying time tended to be increased in CML calves in the period following weaning at 71 days of age. There were no effects of forage or interactive effects of MR level X forage on hourly lying behaviour throughout pre-step down period or any of the post-step down or weaning periods.

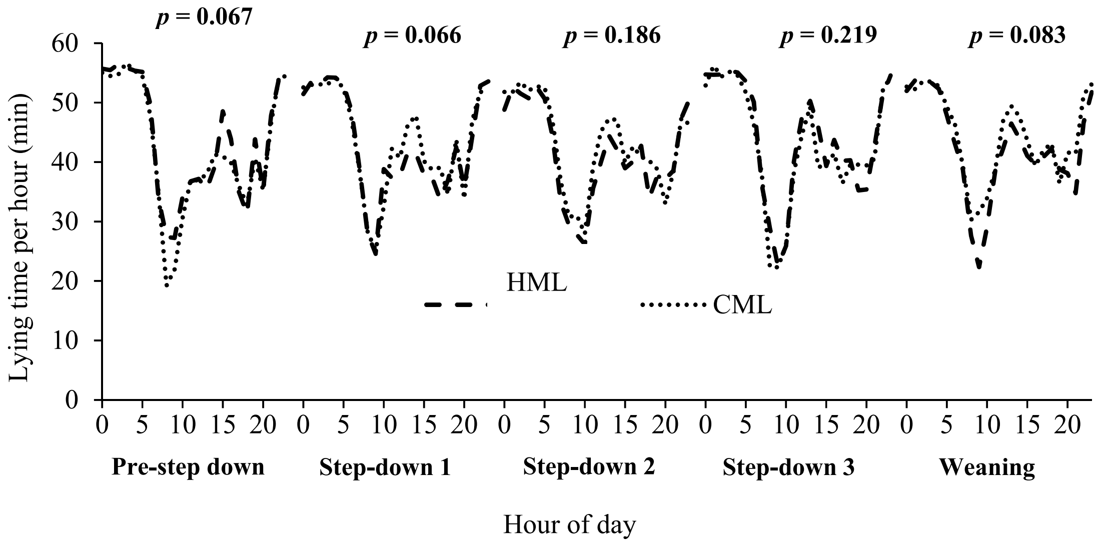

Figure S1. Diurnal lying behaviour of calves offered increased (HML) or conventional (CML) levels of milk replacers over periods of milk step down and weaning. P-values represent the effect of milk replacer feeding level.
